# Condylar constrained knee prosthesis and rotating hinge prosthesis for revision total knee arthroplasty for mechanical failure have not the same indications and same results

**DOI:** 10.1051/sicotj/2021046

**Published:** 2021-09-10

**Authors:** William Barnoud, Axel Schmidt, John Swan, Elliot Sappey-Marinier, Cécile Batailler, Elvire Servien, Sébastien Lustig

**Affiliations:** 1 FIFA Medical Center of Excellence, Orthopaedics Surgery and Sports Medicine Department, Croix-Rousse Hospital, Hospices Civils de Lyon 103 grande rue de la croix rousse 69004 Lyon France; 2 LIBM – EA 7424, Interuniversity Laboratory of Biology of Mobility, Claude Bernard Lyon 1 University 29 Boulevard du 11 Novembre 1918 Lyon France; 3 Univ Lyon, Claude Bernard Lyon 1 University, IFSTTAR, LBMC UMR_T9406 25 Avenue François Mitterand Lyon France

**Keywords:** Total knee arthroplasty, Condylar constrained knee prosthesis, Rotating hinge knee prosthesis, Clinical outcomes, Satisfaction

## Abstract

*Purpose*: This study aimed to evaluate whether there are any differences in outcomes and complication rates between condylar constrained knee (CCK) and rotating hinge knee (RHK) prostheses used for the first revision of total knee arthroplasty (rTKA) after mechanical failure. *Methods*: Sixty-three consecutive non-septic revisions of posterior stabilized implants using 33 CCK and 30 RHK prostheses were included. Clinical evaluation and revision rate were compared between the two groups at two years minimum follow-up. *Results*: The CCK group had significantly better clinical outcomes and satisfaction rates compared to patients with RHK (KSS-knee 70.5 versus 60.7 (*p* < 0.003) and KSS-function 74.9 versus 47.7 (*p* < 0.004) at 3.7 (2.0–9.4) years mean follow-up. Moreover, the clinical improvement was significantly higher for the CCK group concerning the KSS-Knee (+23.9 vs. +15.2 points, *p* = 0.03). The postoperative flexion was significantly better in the CCK group compared to the RHK group (115° vs. 103°, *p* = 0.01). The prosthesis-related complications and the re-revision rate were higher in the RHK group, especially due to patellofemoral complications and mechanical failures. *Conclusions*: CCK prostheses provided better clinical and functional outcomes and fewer complications than RHK prostheses when used for the first non-septic rTKA. CCK is a safe and effective implant for selected patients, while RHK should be used with caution as a salvage device for complex knee conditions, with particular attention to the balance of the extensor mechanism.

## Introduction

Total knee arthroplasty revisions (rTKA) are more complex than primary procedures, with poorer outcomes and higher complications [1]. Major causes of revision are multiple and include infection, aseptic loosening, instability, and stiffness [2].

Major difficulties include managing ligamentous laxity and bone loss, which require an increase in the level of prosthesis constraint [3]. Most rTKAs can be performed using a non-constrained posterior stabilized (PS) implant if the collateral ligaments are intact [4]. However, a rotating hinge knee (RHK) prosthesis is necessary in cases of ligament insufficiency [5]. Condylar constrained knee (CCK) prostheses represent an alternative to RHK in intermediate collateral ligament insufficiency [6].

There is debate as to whether RHK or CCK prostheses provide the most favorable clinical outcomes, complication rate, and survivorship in rTKA [7–14]. Hinged prostheses are generally considered to result in a higher complication rate and lower survivorship compared to other prostheses [15]. However, contemporary RHK designs have decreased aseptic loosening and patellofemoral complications in some studies [16].

This study aimed to evaluate outcomes and postoperative complications at two years minimum follow-up in a consecutive series of patients undergoing a first total revision of a PS prosthesis for mechanical failure using a single CCK or RHK implant. The authors hypothesized that CCK prostheses are associated with better clinical outcomes and a lower rate of postoperative complications than RHK prostheses in the short term.

## Materials and methods

### Patients

A prospectively gathered database of rTKA performed at one institution was retrospectively reviewed, and 325 patients were identified who underwent rTKA between January 2010 and July 2018.

**Figure 1:**
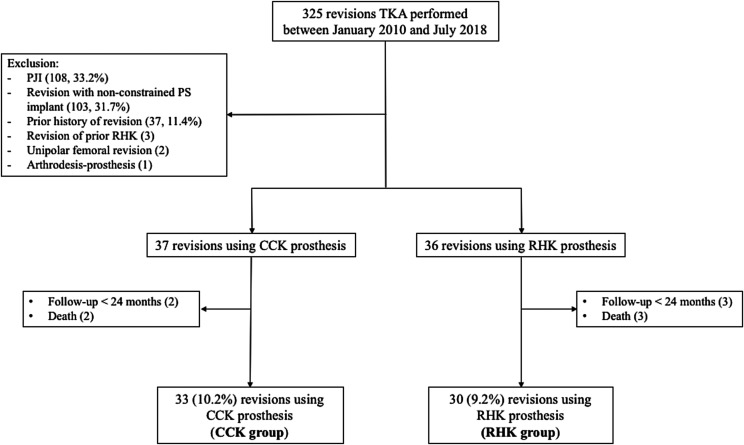
Study flowchart. TKA: Total Knee Arthroplasty; CCK: Condylar Constrained Knee; RHK: Rotating hinge knee; PJI: prosthetic joint infection.

Inclusion criteria were all first rTKA for mechanical failure using CCK or RHK prosthesis, with a minimum follow-up of two years. Exclusion criteria were revisions for prosthetic joint infection (PJI), revisions using a non-constrained PS implant, arthrodesis, primary CCK or RHK prosthesis, unipolar revisions, and patients with prior history of rTKA (Figure 1). Sixty-three patients (63 knees) were retrospectively reviewed, including 20 men and 43 women. There were 33 patients in the CCK group and 30 patients in the RHK group. The mean age was 67 years (range: 47–89). The group demographics are presented in Table 1.

**Table 1:** Comparison of patient demographics by group.

	CCK group (*n* = 33)^a^	RHK group (*n* = 30)^a^	*P* value
Age at surgery (year)	65 ± 10 [47 – 87]	71 ± 8 [55 – 89]	0.008
Follow-up (months)	27.8 ± 5.2 [24 – 51]	33.3 ± 13.9 [24 – 78.8]	0.01
Sex (female)	20/33 (60.6%)	23/30 (76.7%)	n.s.
BMI	30.6 ± 4.1 [24.2 – 42.9]	30.9 ± 7.6 [19.8 – 55]	n.s.
Delay from primary TKA (months)	51.4 ± 61 (5.6 – 228)	49.9 ± 43.6 (2.2 – 137)	n.s.
ASA score			
ASA 1	5 (15.2%)	5 (17.2%)	n.s.
ASA 2	18 (54.5%)	14 (46.7%)	n.s.
ASA 3	10 (30.3%)	11 (37.9%)	n.s.
Indication for revision			
Aseptic loosening	17 (51.5%)	9 (30%)	n.s.
Femorotibial instability	9 (27.3%)	14 (46.7%)	n.s.
Stiffness	3 (9.1%)	4 (13.3%)	n.s.
Patellofemoral instability	2 (6.1%)	3 (10%)	n.s.
Persistent pain	2 (6.1%)	0 (0%)	n.s.
Preoperative flexion ROM (°)	107.3 ± 21 (60 – 135)	99.2 ± 25.9 (30 – 140)	n.s.
Preoperative mFTA (°)	178.4 ± 3.9 [166 – 185]	176.2 ± 8.1 [151 – 185]	n.s.

a Data are presented as mean ± standard deviation [minimum – maximum] or number (proportion).

BMI: body mass index (kg/m^2^); ASA: American Society of Anesthesiologists; mFTA: mechanical femorotibial angle; ROM: Range of motion; n.s.: non-significant.

### Surgery

**Figure 2:**
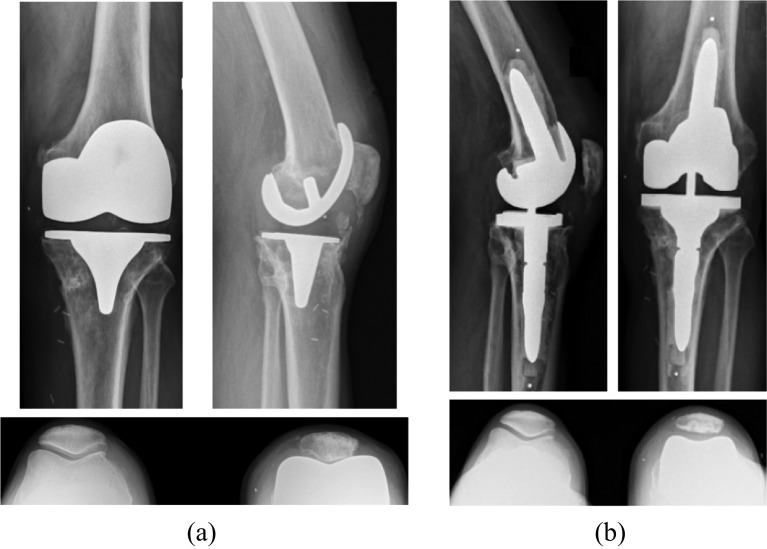
A 75-year-old man with painful TKA secondary to oversized femoral component (A). Revision TKA was performed with a CCK prosthesis using small cemented stems (Triathlon TS – Stryker Orthopaedics^®^) with excellent outcomes at two years follow-up (B).

**Figure 3:**
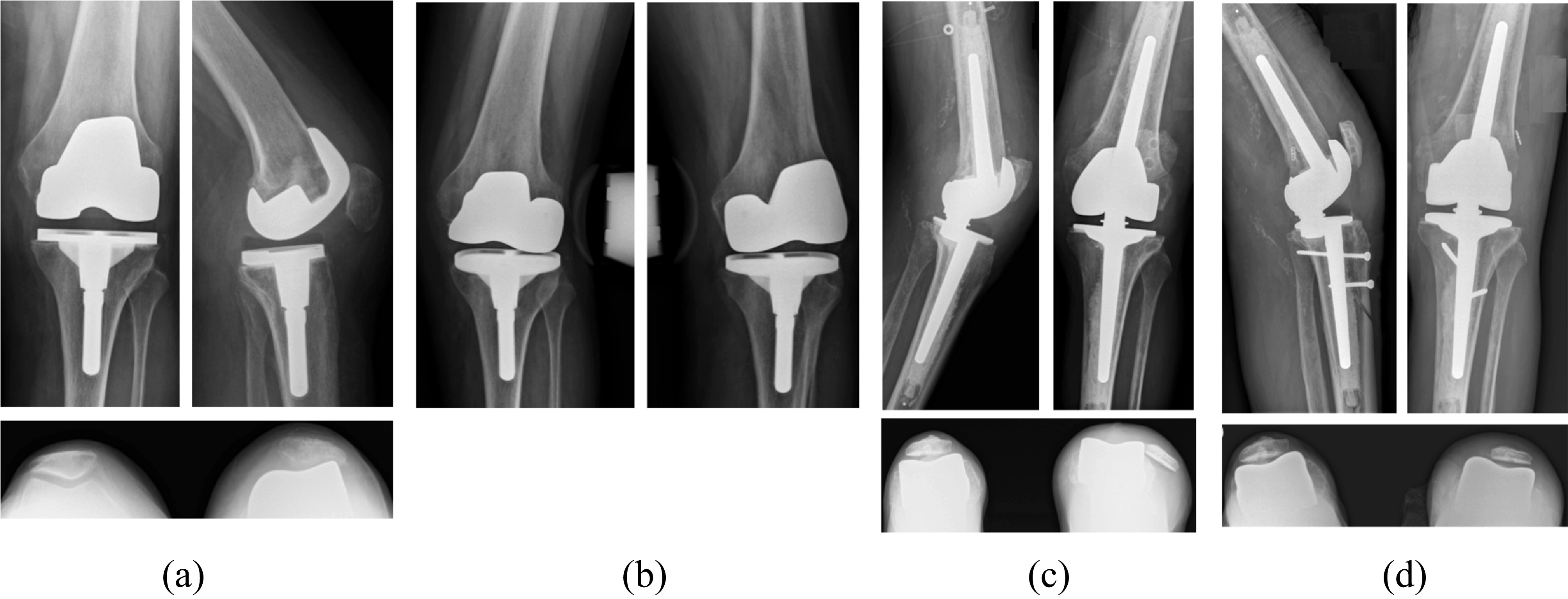
A 74-years-old woman with femorotibial instability of a primary TKA with two prior prosthesis dislocations (A) and (B). A revision TKA with an RHK prosthesis (Rotax – Groupe Lépine^®^) was performed. A recurrent patellar dislocation appears at two months postoperatively (C), requiring further surgery to stabilize the patella: medialization of the anterior tibial tuberosity via osteotomy and medial patello-femoral ligament reconstruction with quadriceps tendon autograft and endo-button fixation through a femoral tunnel (D).

The choice between CCK and RHK prosthesis was preoperatively planned according to the clinical appreciation of the coronal laxity and the radiological investigations with varus-valgus stress X-rays. In cases of minimal (<5°) or moderate coronal laxity (6–9°), a CCK prosthesis was implanted (Triathlon TS – Stryker Orthopaedics^®^, Mahwah, NJ, USA) (Figure 2). In cases of major laxity (>10°) or significant patient subjective instability, an RHK prosthesis was implanted (Rotax – Groupe Lépine^®^, Geney, France) (Figure 3). The Engh and Amneen classification was used to evaluate the severity of bone loss [17]. Long stems were used systematically to augment prosthesis fixation. Metallic augments and cones were used in cases with significant bone loss. There was no significant difference between the two groups regarding preoperative bone loss and the use of metal augments. In all cases, revision implants were fixed with cementation of implant surfaces and stems (Palacos^®^ R + Gentamicin, Heraeus Group, France).

### Evaluation

For both groups, the clinical evaluation score and patient satisfaction were collected at the last consultation with the surgeon before revision and the last follow-up after revision using the Knee Society Score (KSS) knee and function scores [18].

Radiological analysis (standard anteroposterior and lateral knee radiographs, full-length bilateral standing radiograph and patellar axial view radiograph) was performed at the last follow-up to evaluate the mechanical femorotibial angle (mFTA), the incidence of patellar tilt and radiolucent lines greater than 2 mm. Measurement of patellar tilt was made according to the method described by Ls et al. [19] using radiographic patellar axial view at 30° of flexion and was considered significant when tilt measured > 10°.

The complication rate was evaluated at the last follow-up, including all indications of reintervention and all reintervention procedures.

### Ethical approval

All procedures performed in studies involving human participants were by the ethical standards of the institutional and/or national research committee and with the 1964 Helsinki declaration and its later amendments or comparable ethical standards. For this type of study, formal patient consent was not required. The paper was approved by the Institute Review Board. The Advisory Committee on Research Information Processing in the Field of Health (ID-RCB: 2019-A00478-49) approved this study on March 3, 2019.

### Statistical analysis

Statistical analysis was performed using the online software EasyMedStat (http://www.easymedstat.com; Neuilly-sur-Seine, France). Distributions of continuous variables were reported as mean with standard deviation and range. Statistical analysis was performed using the Fischer Test or Mann-Whitney Tests. Categorical variables were compared using a Fisher exact test. Survival analysis was conducted with reintervention as the endpoint. Global survival curves were estimated with Kaplan–Meier model, and the comparison of survivorship between the different initial aetiologies were estimated with log-rank. The level of significance was set at *p* < 0.05 for all tests.

Post-hoc analyses were performed for clinical results. For a power (1 − *β*) at 0.9, the number of patients required to be statistically significant were 11, 28, and 9, respectively for the KSS-total, KSS-Knee, and KSS-Function scores.

## Results

### Clinical outcomes

In both groups, postoperative KSS scores were significantly improved compared to preoperative scores. The difference between the pre and postoperative periods was significant concerning the Knee KSS score (*p* = 0.001 and 0.0001) and the Function KSS score (*p* = 0.001 and 0.01 respectively for the CCK and RHK groups).

Better postoperative clinical outcomes were observed in the CCK group than in the RHK group, with a significant difference in the KSS-Knee (*p* = 0.003) and the KSS-Function (*p* = 0.004). Multivariate analysis concerning postoperative KSS score found a risk factor at −0.8 [−1.65; −0.04] (*p* = 0.03) for age at rTKA.

Postoperative knee flexion was significantly better in the CCK group (115° ± 13° [85°−130°]) compared to RHK group (103° ± 19° [60°–135°]), *p* = 0.01). The flexion improvement was statistically significant in the CCK group (+7.7°, *p* = 0.06) but not in the RHK group (+4.2°, n.s.). Detailed clinical results are summarized in Table 2.

**Table 2:** Comparison of KSS and satisfaction by group.

	CCK group (*n* = 33)^a^	RHK group (*n* = 30)^a^	*P* value
Preoperative KSS			
Knee	46.6 ± 11.7 [12 – 69]	45.6 ± 14.9 [20 – 85]	n.s.
Function	47.3 ± 14.8 [10 – 80]	30.9 ± 19.5 [0 – 68]	0.05
Total	93.9 ± 21.6 [27 – 129]	76.5 ± 28.6 [20 – 135]	0.008
Postoperative KSS			
Knee	70.5 ± 10.2 [36 – 87]	60.7 ± 14.4 [41 – 95]	0.003
Function	74.9 ± 12.5 [40 – 100]	47.7 ± 21.4 [10 – 90]	0.004
Total	145.3 ± 20 [81 – 179]	108.4 ± 32.8 [51 – 169]	0.03
KSS Improvement			
Knee	23.9 ± 14.1 [−21 – 58]	15.2 ± 15.9 [−14 – 51]	0.03
Function	27.6 ± 17.8 [−5 – 60]	16.7 ± 28.1 [−40 – 70]	n.s.
Total	51.5 ± 27.6 [−21 – 98]	31.9 ± 40.2 [−54 – 121]	0.03
Postoperative satisfaction	26/33 (78.8%)	14/30 (46.7%)	0.01

a Data are presented as mean ± standard deviation [minimum–maximum] or number (proportion).

CCK: Condylar Constrained Knee; RHK: Rotating hinge knee; KSS = Knee society score; n.s.: non-significant

### Radiological outcomes

There was no significant difference in mean mFTA between groups before and after revision. In the RHK group, anteroposterior radiographs revealed radiolucent lines in two cases (6.7%). One progressive around the tibial component at the bone-cement interface led to a bipolar revision 23 months after rTKA for aseptic loosening. The second case was observed around the femoral implant and was not progressive after further follow-up. No radiolucent lines were observed in the CCK group, but the statistical difference with RHK was non-significant (*p* = 0.5). All metal augments, stems and metaphyseal cones used were well fixed radiologically at the final follow-up. The incidence of postoperative patellar tilt > 10° was significantly higher with 10/30 (30%) in the RHK group versus 4/33 (12.1%) in the CCK group (*p* = 0.04).

### Complications and failure of revision

The reported number of prosthesis-related complications at the last follow-up was higher in the RHK group. In this group, an abnormality of the extensor mechanism was most common (*p* = 0.04), including five cases of patellofemoral instability (Figure 3). Of the five patients with patellar dislocation, three patients were declined for further intervention because of an unfavorable risk-benefit ratio.

The number of surgical re-interventions after revision for any prosthetic complication was higher in the RHK group compared to the CCK group (*p* = 0.04). Furthermore, only patients within the RHK group underwent further revision requiring an implant change. It was due to many mechanical failures (5/6, 83.3%) within two years. The indications were aseptic (3) and septic (1) loosening, patella dislocation (1), and femorotibial instability with RHK dislocation (1). Details of complications related to the revision prosthesis are summarized in Table 3.

**Table 3:** Complications related to the revision prosthesis at two years.

	CCK group	RHK group	*P* value
Complication^a^	4/33 (12.1%)	12/30 (40%)	0.04
Type of complication^b^			
Acute PJI	2	1	n.s.
Aseptic loosening	0	3	n.s.
Septic loosening	0	1	n.s.
Extensor mechanism	0	6	0.04
Stiffness	2	1	n.s.
Femorotibial instability	0	1	n.s.
Re-intervention^b^			
DAIR	2	1	n.s.
Arthrolysis	2	2	n.s.
Extensor mechanism intervention	0	2	n.s.
Re-revision with change of implant	0	6	0.008
Total	4 (12.1%)	11 (36.7%)	0.04

a Data are presented as number of patients (proportion).

b Complications are presented in number of occurrences. Two patients of RHK group had several consecutive complications.

PJI: prosthetic joint infection; DAIR: debridement, antibiotics, and implant retention.

**Figure 4:**
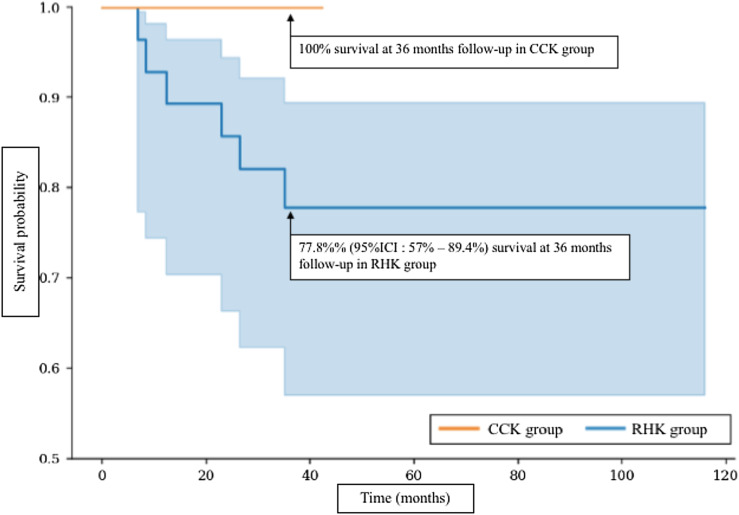
Re-revision-free survival curves comparing the CCK group and RHK group.

The re-revision-free survival rate at 36 months was 100% for the CCK group and 77.8% for the RHK group (Figure 4).

## Discussion

The most important finding of this study was the better clinical and functional outcomes for patients who underwent the first revision of PS prosthesis for mechanical reasons using a CCK prosthesis compared to RHK prosthesis, at short-term follow-up of 2 years after revision even if the indication were not the same. Moreover, the clinical improvement was significantly higher for the CCK group regarding the KSS-Knee score (Table 2). In these results, better postoperative KSS scores in the CCK group were related to a significantly higher proportion of satisfied patients.

The literature reports contrasting postoperative clinical and functional outcomes between CCK and RHK in revision of total knee arthroplasty. Four authors [7–10] using KSS, Oxford Knee Score, Hospital for Special Surgery (HSS) knee score, or SF-36 showed that increased constraint using RHK was associated with poorer outcomes than CCK in the rTKA. Lim *et al.* [8] found, in a series of patients who received a CCK or RHK prosthesis for a primary or revision procedure, better KSS-function and SF-36 scores for the CCK patients, with a greater proportion of satisfied patients at mid-term follow-up of two years. These findings are similar to the results of this study and those reported by Luttjeboer *et al.* [9], who found better results concerning pain and patient satisfaction in the CCK group.

In contrast, several studies [11–14] found no statistically significant differences in the clinical outcomes and implant failure rate. For example, Fuchs *et al.* [12] followed 26 rTKA using CCK or RHK for an average of 20.4 months and reported no difference of KSS, Hospital for Special Surgery score (HSS), Tegner score, and Visual Analogue Score. Only patients who underwent multiple revisions for aseptic loosening and had received a CCK implant had significantly lower scores in general health perception, social functioning, general mental health, and mental component summary.

A second important finding of this study was the greater flexion range of motion (ROM) for the CCK group compared to the RHK group. Results regarding flexion ROM in other studies comparing CCK versus RHK for rTKA are conflicting. Fuchs *et al.* [12] reported lower flexion ROM in RHK versus CCK (96.5° vs. 107.5° *p* < 0.05). However, one author reported no significant difference between the two implants concerning flexion [14]. In contrast, Hossain *et al.* [7] found a higher flexion ROM for RHK as compared to CCK in a series of revisions (111.7° vs. 106.1°, *p* < 0.0001).

In this series, the rate of prosthesis-related complications was significantly lower in the CCK group than in the RHK group (Table 3). This study did not report any cases of loosening or subsequent revision of a CCK prosthesis at two years after the primary revision. Similar results are reported by Stevens *et al.* [20] using the same implant (Triathlon TS – Stryker Orthopaedics^®^) in a series of 100 rTKA with a 2-years survival rate of 96%.

This study found a relatively large number of complications in the RHK group involving 40% of patients (Table 3). These results are consistent with other studies. A published review of the literature [21] concerning RHK prostheses for rTKA reported a complication rate ranging from 9.2% to 63%. In particular, these results regarding complications are comparable to those of Springer *et al.* [22], who showed, in a series of 69 RHK prosthesis including 57 revisions, that complications related to the extensor mechanism was the third most frequent complication and involved 13% of patients. Furthermore, this study reported more patients with patellar tilt > 10° in the RHK group. Multiple reasons could explain patellar tilt, such as resurfacing technique, surgical approach, femoral implant design, lateral retinacular release, or implant rotation. This present study did not attempt to identify a cause of patellar tilt. This study hypothesized that the more extensive surgical approach, the condition of the soft tissue, the implant positioning, and rotation, are potential reasons for patellofemoral complications with these RHK prostheses.

In this study, the six cases requiring further implant revision involved RHK prostheses (20%), statistically more than the CCK group and due to mechanical reasons. Mechanical complications reported in the literature with RHK prostheses are frequent [21]. Regarding re-revisions, these results are comparable to Joshi and Navarro-Quilis [23], who reported 19/78 RHK having prosthesis-related failure with a mean delay from a revision of 20 months. Of these 19 knees, ten required an implant change, including six for loosening.

A recent meta-analysis [24], including 12 studies comparing RHK with CCK, reported significantly better pain and function scores for CCK groups, but no difference in ROM or survival rate at five and ten years. Furthermore, the reason for the revision was identified as the source of heterogeneity for the survival rates. The present study is the first comparing CCK and RHK specifically for the first revision of posterior stabilized implants and a non-septic etiology. Most existing published studies comparing CCK and RHK include heterogeneous groups of patients combining septic and aseptic indications [7, 12–14], tumoral indications [11], and first and subsequent revision [8, 12]. Moreover, the revised implant is often not specified. Luque *et al.* [25] found that septic loosening has a statistically significant association with poor postoperative outcomes after revision compared to aseptic loosening. Moreover, prosthetic knees that had already undergone several revisions often have more altered preoperative conditions than a primary revision, which can result in poorer postoperative outcomes. All these factors may explain the contrasting results in the literature and our rationale to specifically exclude patients from our study with prior history of rTKA, revisions not involving PS implants, and revision for a septic etiology to make the groups comparable.

This study has several limitations. Firstly, it is a retrospective analysis with a limited follow-up and a limited number of patients. Although the follow-up time is relatively short, Schmidt *et al.* [26], showed in a retrospective study that 90.1% of rTKA failures occur within the first two years after revision. Secondly, the two groups could not be matched based on age and cause of revision, but the other inclusion criteria were intentionally strict to allow us to have two comparable groups. Thirdly, although there was no significant preoperative difference between the two groups except for age. The use of additional functionality and quality of life scores could thus have shown poorer preoperative conditions for the RHK group and, poorer postoperative outcomes. Therefore, a confusion bias cannot be excluded with regard to outcome scores.

## Conclusion

This study suggests that given the better clinical and functional outcomes of CCK prostheses with less complications than RHK prostheses, it is preferable to use a CCK design if increased constraint is required and preoperative conditions, such as preoperative moderate laxity and bone loss allow it. Given the high complication rate in these results for RHK, it is, therefore, advisable to use the RHK with caution as a salvage implant for complex knee conditions with particular attention to the balance of the extensor mechanism.

## Conflict of interest

No benefits in any form have been received or will be received from a commercial party related directly or indirectly to the subject of this article.

## Funding

This research did not receive any specific funding.

## Ethical approval

The paper was approved by the Institute Review Board. The Advisory Committee on Research Information Processing in the Field of Health (ID-RCB: 2019-A00478-49) approved this study on March 3, 2019.

## Authors contribution

All the authors have contributed to this study.
